# Metabolic reprogramming in the post-metastatic tumor microenvironment: multi-omics insights into determinants of immunotherapy response

**DOI:** 10.3389/fimmu.2025.1742855

**Published:** 2026-01-05

**Authors:** Mengxi Li, Tingting Wang, Zhenwang Zhang, Yuxi Dongye

**Affiliations:** 1 School of Nuclear Technology and Chemistry & Biology, Hubei University of Science and Technology, Xianning, Hubei, China; 2 Department of Dermatovenereology, West China Hospital, Sichuan University, Chengdu, China; 3 Laboratory of Dermatology, Clinical Institute of Inflammation and Immunology, Frontiers Science Center for Disease-related Molecular Network, West China Hospital, Sichuan University, Chengdu, China; 4 Department of Breast Surgery, Jinan Third People’s Hospital, Jinan, Shandong, China

**Keywords:** immunotherapy resistance, metabolic reprogramming, multi-omics integration, precision oncology, tumor immune microenvironment

## Abstract

Metabolic reprogramming has emerged as a central determinant of immune modulation in the post-metastatic tumor immune microenvironment (TIME). Alterations in glycolysis and lactate accumulation, lipid metabolic rewiring, metal-dependent cell death pathways such as ferroptosis and cuproptosis, and the tryptophan–IDO1–kynurenine axis collectively contribute to an immunosuppressive niche that drives tumor progression and therapeutic resistance. These metabolic shifts are not isolated events but are intricately connected with immune-regulatory networks, profoundly influencing the efficacy of immunotherapy. Advances in multi-omics technologies—including metabolomics, proteomics, single-cell sequencing, and spatial omics—have provided unprecedented resolution to decode these complex interactions, enabling the identification of predictive biomarkers, delineation of metabolic–immune signatures, and discovery of therapeutic vulnerabilities. Integrating these multi-layered datasets has paved the way for precision medicine strategies that tailor immunotherapy to patient-specific metabolic and immune contexts. Therapeutically, combining metabolic inhibitors with immune checkpoint blockade, exploiting ferroptosis or cuproptosis to enhance tumor immunogenicity, or modulating amino acid metabolism to reverse immune tolerance are promising strategies to overcome resistance and expand patient benefit. Looking forward, the integration of multi-omics-guided biomarkers, AI-driven analytics, and advanced delivery systems such as nanoparticles and engineered exosomes will accelerate the translation of these insights into clinical practice. Decoding the metabolism–immunity crosstalk through multi-omics not only advances our understanding of metastatic cancer biology but also paves the way for next-generation personalized and adaptive therapies that promise to enhance immunotherapy efficacy, prolong survival, and improve the quality of life for patients with advanced cancers.

## 1 Introduction

In contemporary oncology, therapeutic strategies increasingly extend beyond directly eliminating cancer cells to actively reshaping their surrounding microenvironment (1, 2). Conceptually, this shift has been framed as targeting either “the seed” or “the soil,” emphasizing that durable clinical responses often require simultaneous control of malignant cells and their supportive niches (3). Within this framework, metabolism–immunity crosstalk has emerged as a central regulatory axis: metabolic alterations in tumors and stromal cells not only fuel growth and metastasis but also reprogram immune surveillance and determine the success or failure of systemic therapies, including immune checkpoint blockade (4, 5). Tumor metastasis represents a critical stage in cancer progression and is the primary cause of mortality among patients with advanced malignancies. Following metastatic dissemination, tumor cells not only adapt to new organ-specific niches but also profoundly reshape the post-metastatic tumor immune microenvironment (TIME) (6–9). This microenvironment is characterized by a dynamic interplay among malignant cells, stromal components, and infiltrating immune cells, which collectively determine the success of metastatic colonization, immune evasion, and therapeutic resistance (10–13). Compared to primary tumors, post-metastatic TIME displays higher cellular heterogeneity, altered cytokine networks, and more profound metabolic rewiring, creating a highly immunosuppressive milieu that poses major challenges to effective therapy.

A hallmark of this reprogramming is the bidirectional interplay between metabolism and immunity (14, 15). Tumor cells undergoing metabolic rewiring—such as heightened glycolysis with lactate accumulation, altered lipid metabolism, and perturbed metal-dependent cell death programs (ferroptosis and cuproptosis)—can directly suppress effector T-cell function, expand regulatory T cells (Tregs), and drive macrophage polarization toward immunosuppressive phenotypes (16–18). Conversely, immune cells in the TIME must adapt to nutrient deprivation and metabolic stress, and impaired metabolic fitness contributes to T-cell dysfunction and weakened anti-tumor immunity (19–21). The tryptophan–IDO1–kynurenine axis exemplifies how metabolic intermediates act as immune checkpoints, dampening cytotoxic immune responses and facilitating tumor immune escape (22–24). This intricate metabolism–immunity crosstalk underscores the necessity of studying metabolism not in isolation but as a central determinant of immunotherapy response.

The emergence of multi-omics technologies has provided unprecedented opportunities to unravel the complex metabolic–immune interactions that shape the TIME after metastasis (25–28). Each omics layer captures a distinct dimension of biological regulation, and when integrated, they provide a comprehensive picture that transcends the limitations of single-modality analyses. Metabolomics allows direct detection and quantification of metabolites, providing functional readouts of ongoing biochemical activities (25–28). By profiling metabolites such as lactate, kynurenine, and lipid intermediates, metabolomics has revealed metabolic signatures strongly associated with immune suppression, tumor progression, and resistance to immune checkpoint inhibitors. These metabolite-based biomarkers not only reflect tumor cell activity but also capture immune cell dysfunction, offering valuable indicators for patient stratification and therapeutic monitoring. Proteomics and phosphoproteomics add another critical layer by characterizing the abundance and modification status of proteins that regulate both metabolism and immunity. Post-translational modifications—such as phosphorylation, acetylation, ubiquitination, and lipidation—profoundly influence the stability and activity of metabolic enzymes and immune regulators (29–32). For instance, phosphorylation of glycolytic enzymes can accelerate lactate production, whereas ubiquitination of immune checkpoint proteins like PD-L1 modulates their stability and function (33, 34). Thus, proteomics enables the mapping of metabolism–signaling–immunity networks, linking biochemical flux to immune cell behavior and therapeutic outcomes. Single-cell and spatial multi-omics represent a transformative advance by resolving cellular heterogeneity and tissue architecture within metastatic niches (35–37). Single-cell transcriptomics coupled with metabolomic profiling has revealed distinct subsets of metabolically exhausted CD8^+^ T cells, lipid-accumulating macrophages, and immunomodulatory fibroblasts. Meanwhile, spatial transcriptomics and imaging mass spectrometry have highlighted metabolic gradients within tumor lesions, showing how lactate-rich zones or cholesterol-enriched microdomains correlate with immune exclusion and therapy resistance. These insights underscore the importance of considering both cell-type specificity and spatial context when studying metabolic reprogramming. By integrating multi-omics datasets, researchers can construct a panoramic view of metabolic rewiring events that dictate immune dynamics and treatment outcomes in advanced cancer. Systems-level approaches, such as network analysis and machine learning, enable the identification of convergent pathways that drive immune suppression and highlight vulnerabilities that may be exploited therapeutically. Importantly, such integrative analyses lay the groundwork for precision oncology, where metabolic–immune signatures can guide personalized immunotherapy regimens, predict therapeutic response, and inform rational design of combination strategies.

This review synthesizes emerging evidence that metabolic reprogramming is a key driver of immune dysfunction in the post-metastatic TIME and a major determinant of response to immunotherapy. A central knowledge gap is how spatially and cell-type–specific metabolic programs within metastatic lesions orchestrate immune evasion and therapy resistance, and how multi-omics can be leveraged to resolve and therapeutically exploit these programs. We discuss representative immunometabolic axes—including lactate and lipid metabolism, tryptophan–IDO1–kynurenine signaling, and metal-dependent cell death programs (ferroptosis/cuproptosis)—and highlight multi-omics evidence that links these pathways to immune states and clinical outcomes. Finally, we outline translational opportunities and challenges for multi-omics–guided metabolism–immunity combination strategies, emphasizing biomarker-driven patient stratification and rational co-targeting paradigms for precision immunotherapy.

## 2 Metabolic reprogramming in the post-metastatic tumor microenvironment

Metabolic reprogramming is a defining hallmark of metastatic tumor progression, enabling cancer cells to adapt to new organ environments while reshaping the surrounding immune landscape. In the post-metastatic TIME, tumor cells, cancer-associated fibroblasts (CAFs), and immune cells establish a bidirectional metabolic crosstalk that fosters immune evasion and therapeutic resistance (38–41). Recent multi-omics studies have revealed profound alterations across glycolysis, lipid metabolism, metal-dependent cell death pathways, and amino acid catabolism, highlighting these processes as determinants of immunotherapy response.

### 2.1 Lactate metabolism and immunosuppression

One of the most studied hallmarks of cancer metabolism is the Warburg effect, whereby tumor cells preferentially rely on aerobic glycolysis even in the presence of sufficient oxygen. This metabolic switch results in the excessive production and accumulation of lactate in the TIME (42–44). Elevated lactate not only acidifies the extracellular milieu, impairing the cytotoxic function of CD8^+^ T cells and natural killer (NK) cells, but also reshapes the stromal and immune compartments to favor tumor progression. A major contributor to this process is the activity of cancer-associated fibroblasts (CAFs), which undergo the so-called “reverse Warburg effect.” In this state, CAFs increase glycolytic flux and export large amounts of lactate via monocarboxylate transporters (MCT1/MCT4) (45–48). This continuous efflux of lactate generates a nutrient-deprived and immunosuppressive microenvironment. Accumulating evidence reveals that lactate does not merely acidify the TIME but functions as an active signaling metabolite that directly upregulates PD-L1 expression on tumor cells. Mechanistically, lactate stabilizes HIF-1α under both hypoxic and normoxic conditions by inhibiting prolyl hydroxylases (PHDs), thereby preventing HIF-1α degradation. Activated HIF-1α binds to hypoxia-responsive elements (HREs) in the PD-L1 promoter, transcriptionally enhancing its expression. In parallel, lactate induces NF-κB activation through the GPR81–cAMP axis, which further promotes PD-L1 transcription (49). Lactate-mediated histone lysine lactylation (Kla) has also emerged as a novel epigenetic mechanism that enhances chromatin accessibility at the PD-L1 locus, facilitating transcriptional activation (50). Together, these pathways establish a lactate–HIF-1α/NF-κB–PD-L1 axis that strongly contributes to immune escape and ICI resistance in metastatic tumors. As a consequence, lactate accumulation triggers a cascade of immunomodulatory effects: T-cell exhaustion: chronic exposure to high lactate concentrations reduces T-cell receptor (TCR) signaling and decreases IFN-γ production, driving effector T cells into a dysfunctional state (51). Macrophage polarization: lactate functions as a signaling metabolite that promotes the skewing of tumor-associated macrophages (TAMs) toward the M2 immunosuppressive phenotype, which supports angiogenesis, tissue remodeling, and tumor growth. NK cell impairment: lactate directly suppresses NK cell cytotoxicity by disrupting mitochondrial metabolism, thereby reducing their capacity to eliminate tumor cells (52–54). Recent multi-omics studies have shed light on the molecular underpinnings of lactate-mediated immune suppression. Metabolomics profiling consistently demonstrates elevated lactate levels in metastatic niches, while transcriptomics and proteomics analyses reveal enrichment of lactate-related gene signatures—including LDHA overexpression, MCT4 upregulation, and HIF1α activation—which correlate with poor prognosis and resistance to immune checkpoint inhibitors (ICIs) (55, 56). For example, integrative analyses combining metabolomics and RNA-seq showed that lactate accumulation is strongly associated with PD-L1 upregulation on tumor cells and concomitant reduction of T-cell infiltration, providing a direct mechanistic link between altered metabolism and immune escape (57). Furthermore, spatially resolved multi-omics analyses suggest that lactate-enriched regions within tumor lesions are associated with immune “cold” niches, characterized by reduced infiltration of cytotoxic CD8^+^ T cells and relative enrichment of immunosuppressive cell populations (58). Together, these observations indicate that lactate metabolism is spatially heterogeneous across tumors and may contribute to the formation of localized metabolic microenvironments that shape immune cell localization and functional states. Therapeutically, targeting lactate metabolism has emerged as a promising strategy to overcome immunosuppression. Inhibition of LDHA or blockade of MCT transporters can restore T-cell cytotoxicity and enhance responsiveness to PD-1/PD-L1 blockade in preclinical models. Additionally, buffering extracellular acidity through systemic alkalinization strategies or nanoparticle-mediated lactate scavenging has shown potential to reprogram the TIME toward a more immunostimulatory state. Ongoing efforts are focused on integrating lactate-targeting therapies with immunotherapy, with the aim of sensitizing resistant tumors and expanding the proportion of patients who benefit from ICIs. Beyond lactate and acidosis, metastatic lesions frequently exhibit broader nutrient and membrane remodeling, making lipid metabolism a second major source of immunoregulatory cues in the TIME.

### 2.2 Lipid metabolism and immune modulation

**Figure 1:**
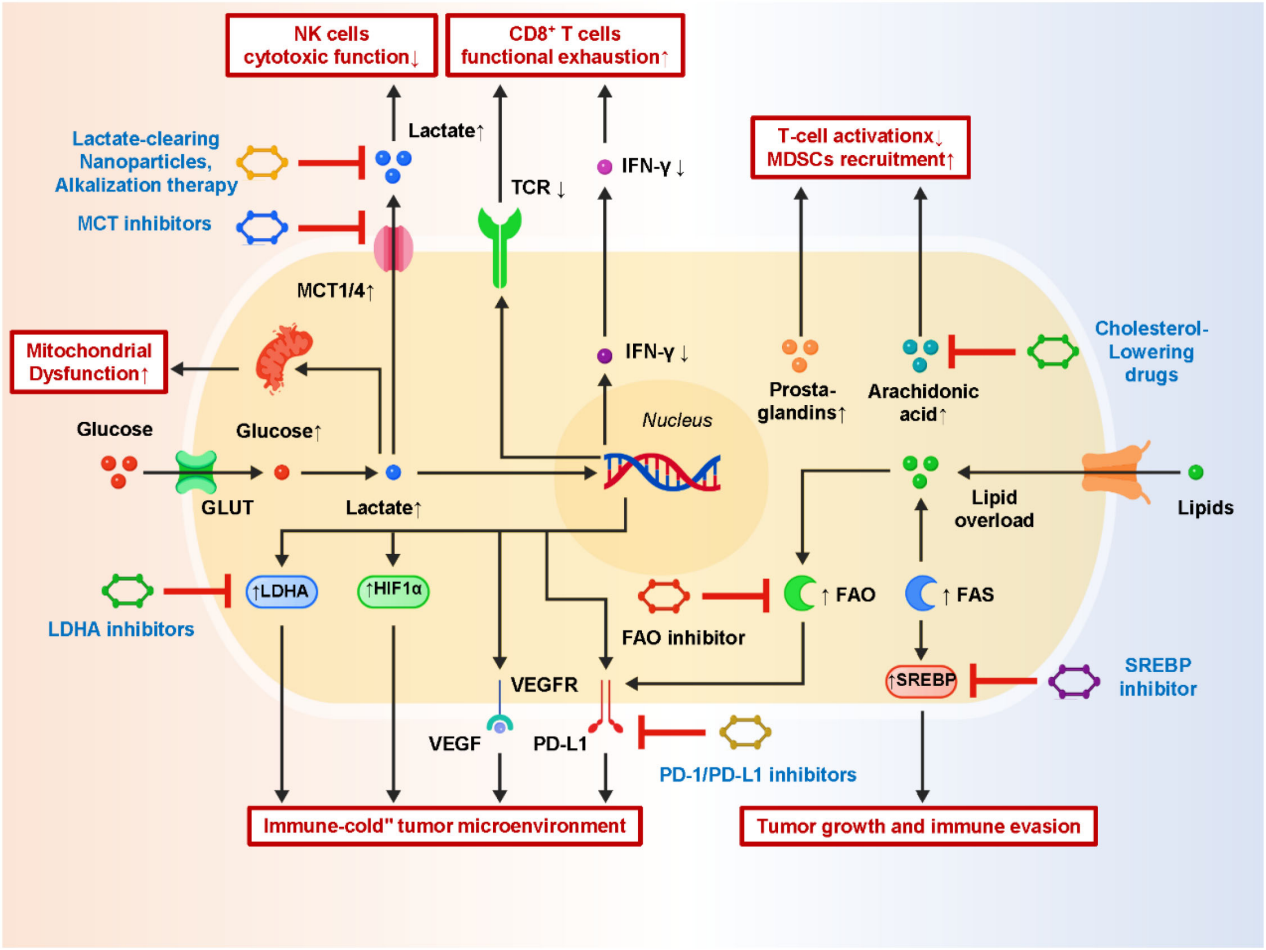
Metabolic rewiring of lactate and lipid pathways drives tumor immune suppression.

Dysregulated lipid metabolism represents another hallmark of the post-metastatic TIME. Unlike normal tissues, which maintain balanced lipid synthesis and degradation, metastatic tumor cells exhibit enhanced fatty acid synthesis (FAS) and fatty acid oxidation (FAO) to support their proliferative and survival needs (59–61). This dual rewiring ensures sufficient production of membrane lipids, signaling molecules, and energy substrates. Importantly, these lipid metabolic adaptations extend beyond tumor-intrinsic functions, directly shaping immune responses and influencing the efficacy of immunotherapy. In tumor cells, elevated FAO activity has been mechanistically linked to the stabilization and upregulation of PD-L1, thereby diminishing T-cell cytotoxicity and attenuating responses to immune checkpoint blockade (ICB) (62). Enhanced lipid biosynthesis, particularly through sterol regulatory element-binding proteins (SREBPs), also drives oncogenic signaling and immune evasion. Moreover, lipid-derived mediators such as prostaglandins and eicosanoids contribute to the establishment of an immunosuppressive microenvironment by suppressing T-cell activation and recruiting myeloid-derived suppressor cells (MDSCs) (63–65). Within immune cells, abnormal lipid metabolism further exacerbates immunosuppression. CD8^+^ T cells with excessive lipid uptake, particularly cholesterol and long-chain fatty acids, undergo mitochondrial stress, leading to metabolic exhaustion and impaired effector function. Similarly, regulatory T cells (Tregs) rely heavily on FAO for survival and expansion, giving them a metabolic advantage in lipid-rich niches. In antigen-presenting cells, lipid accumulation is equally detrimental: dendritic cells (DCs) overloaded with lipids lose their ability to effectively process and present tumor antigens, thereby weakening the priming of anti-tumor T-cell responses (66–68). These observations highlight how tumor-driven lipid alterations reprogram not only cancer cells but also the immune landscape. Spatial multi-omics studies have provided critical insights into the heterogeneity of lipid metabolism across metastatic lesions. Spatial transcriptomics coupled with lipidomics has revealed cholesterol-rich microdomains within tumors, which correlate with reduced T-cell infiltration and altered myeloid cell composition (69–71). Such spatially defined lipid niches act as metabolic barriers, creating localized immune “cold zones” where anti-tumor immunity is profoundly suppressed. Beyond cholesterol, mapping of phospholipids and triglycerides across tissue sections has demonstrated unique lipid landscapes associated with immune exclusion in metastatic cancers such as melanoma and hepatocellular carcinoma. The therapeutic implications of these findings are significant. Inhibitors targeting key lipid metabolic regulators, such as SREBP inhibitors, FAO inhibitors (e.g., etomoxir), and cholesterol-modulating drugs, have shown potential to reprogram the TIME toward a more immunostimulatory state. Combination strategies are being actively explored: for instance, suppressing FAO to reduce PD-L1 expression and thereby enhance responsiveness to PD-1/PD-L1 blockade, or using statins to lower cholesterol accumulation and restore T-cell infiltration. Moreover, multi-omics-derived lipid signatures are being investigated as biomarkers for predicting patient response to immunotherapy and guiding precision treatment strategies. Together, these insights emphasize that lipid metabolism is not a passive bystander but an active determinant of immune regulation in the post-metastatic TIME. By integrating lipidomics, proteomics, and spatial transcriptomics, researchers are beginning to define the lipid–immune networks that underlie resistance to immunotherapy, opening new avenues for therapeutic intervention. **Figure 1** depicts how lactate accumulation and altered lipid metabolism synergistically remodel the TIME into an immune-excluded and immunosuppressive state. Notably, lipid-derived mediators can function as signaling ligands that interface with immune checkpoints and kinase cascades, helping explain why metabolic cues may reshape immune signaling networks and create targetable vulnerabilities in immunotherapy. Building on this signaling-centric view, we next discuss metal-dependent cell death programs—ferroptosis and cuproptosis—as a complementary class of metabolic liabilities that can modulate immunogenicity and therapy response.

### 2.3 Ferroptosis and cuproptosis in TIME

**Figure 2:**
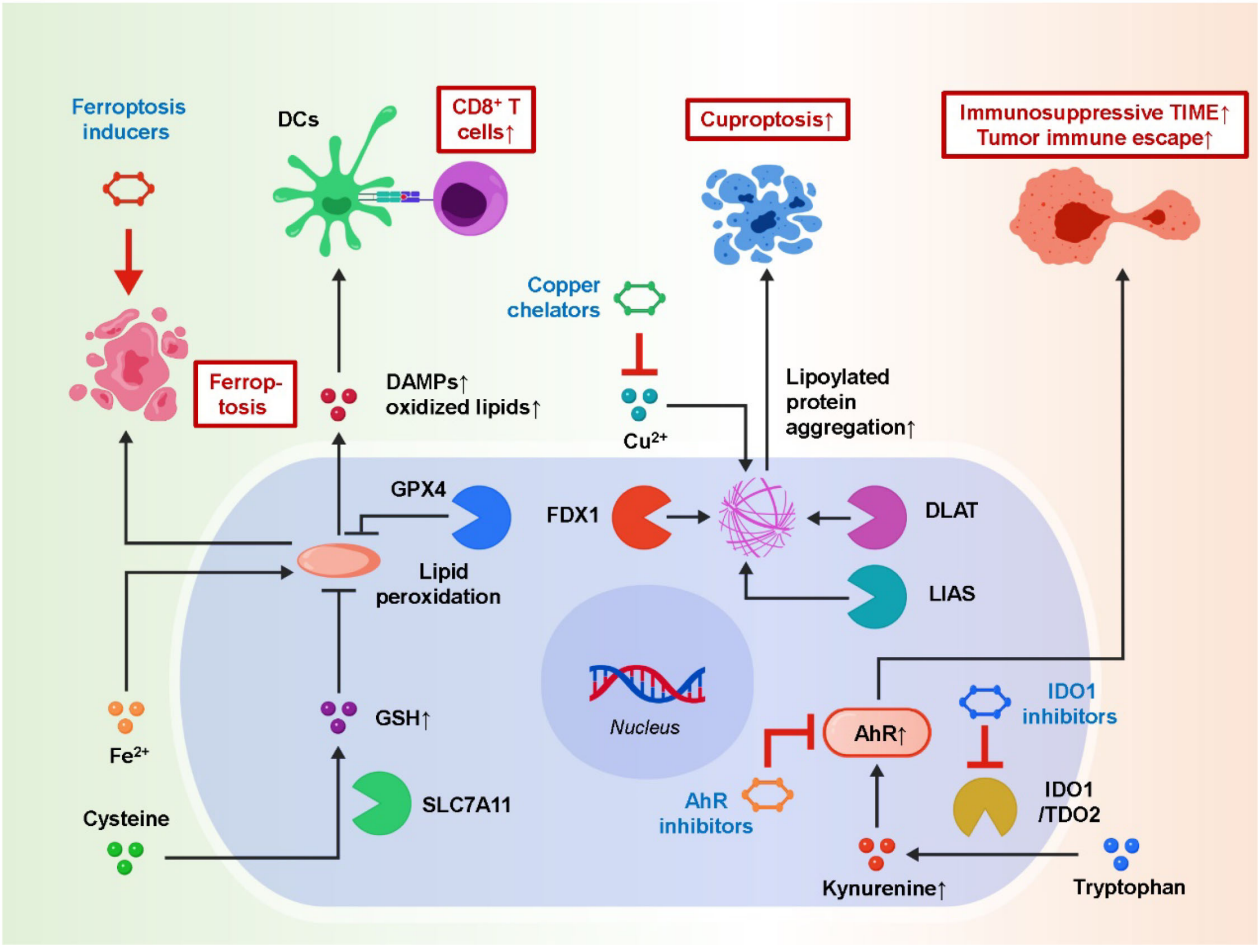
Ferroptosis, cuproptosis, and tryptophan–IDO1–kynurenine pathway cooperatively shape tumor immune escape and ICI resistance in the TIME.

Having discussed metabolite-driven immunosuppression, we next shift to metabolic vulnerabilities that shape immunogenic cell death in the post-metastatic TIME. In contrast to lactate-, lipid-, and tryptophan-derived metabolites that primarily impose extrinsic constraints on immune function, ferroptosis and cuproptosis represent intracellular stress responses that determine how tumor and immune cells die, what danger signals are released, and how antigen-presenting and effector programs are subsequently engaged (72, 73). Importantly, these death programs do not operate in isolation; emerging evidence suggests that metabolic stress can be wired into immune signaling cascades and reveal actionable immunotherapy nodes, consistent with recent efforts to identify new immune targets and signaling pathways for cancer immunotherapy. Ferroptosis, an iron-dependent form of regulated cell death caused by the accumulation of lipid peroxides, has emerged as a pivotal yet paradoxical process in tumor immunity. On the one hand, ferroptotic tumor cells release danger-associated molecular patterns (DAMPs), lipid metabolites, and oxidized phospholipids that can function as immunogenic signals, activating dendritic cells and promoting CD8^+^ T-cell priming (74, 75). This immunostimulatory effect positions ferroptosis as a potential ally in augmenting immunotherapy. On the other hand, excessive ferroptosis in immune cells—including CD8^+^ T cells and natural killer (NK) cells—compromises their persistence, mitochondrial fitness, and effector functions, ultimately weakening the anti-tumor immune response. The balance of ferroptosis is tightly regulated by molecules such as glutathione peroxidase 4 (GPX4) and SLC7A11 (system Xc^-^ transporter). GPX4 detoxifies lipid peroxides, protecting both tumor and immune cells from ferroptotic damage, whereas SLC7A11 maintains cystine uptake and glutathione biosynthesis, providing a metabolic safeguard (76–80). Dysregulation of these pathways in metastatic niches creates conditions where tumor cells resist ferroptosis while immune cells remain vulnerable, reinforcing immune suppression. Cuproptosis, a newly discovered form of cell death, adds another dimension to metabolic–immune regulation. It is triggered by copper-dependent aggregation of lipoylated proteins within the tricarboxylic acid (TCA) cycle, leading to proteotoxic stress and cell death (81). The mitochondrial reductase FDX1 has been identified as a critical mediator of this process. Overexpression of FDX1 enhances sensitivity to copper ionophores, leading to tumor cell death, while aberrant copper homeostasis can also impair the survival and function of immune cells (82–84). Accumulating evidence suggests that copper-rich metastatic niches may foster tumor growth and suppress T-cell activity, hinting at a dual role for copper metabolism in tumor–immune dynamics. Recent multi-omics approaches have begun to unravel these metal-dependent processes. Proteogenomic studies have identified ferroptosis-related gene signatures that predict responsiveness to ICB. Single-cell transcriptomics has revealed heterogeneity in ferroptosis sensitivity, showing that subsets of tumor cells are ferroptosis-prone while immune cells exhibit variable susceptibility depending on their activation state. Similarly, integrated transcriptomic–metabolomic analyses have uncovered cuproptosis-associated gene networks (e.g., FDX1, DLAT, LIAS) that correlate with immunotherapy outcomes in melanoma, lung cancer, and hepatocellular carcinoma (81, 85–87). These findings suggest that ferroptosis and cuproptosis are not merely cell death programs but central regulators of the TIME. Therapeutically, modulating ferroptosis and cuproptosis presents exciting opportunities. Ferroptosis inducers (e.g., erastin, RSL3) have been shown to enhance antigen presentation and synergize with PD-1/PD-L1 blockade in preclinical models. Conversely, protecting T cells from ferroptosis through GPX4 stabilization may enhance their persistence during ICB therapy. In the context of cuproptosis, copper ionophores (e.g., elesclomol) and copper chelators are under investigation as modulators of tumor growth and immunity. Importantly, multi-omics biomarkers may enable patient stratification to identify those most likely to benefit from ferroptosis- or cuproptosis-targeted interventions. Together, ferroptosis and cuproptosis illustrate how metal-dependent metabolic vulnerabilities intersect with immune regulation in the post-metastatic TIME. By leveraging multi-omics technologies to decode these pathways, new therapeutic strategies may emerge to fine-tune the balance between promoting immunogenic tumor cell death and preserving immune cell fitness, ultimately improving the efficacy of immunotherapy. **Figure 2** illustrates how metal-dependent cell death pathways (ferroptosis and cuproptosis) and the tryptophan–IDO1–kynurenine metabolic axis synergistically facilitate tumor immune escape. While ferroptosis and cuproptosis highlight how metabolic stress governs immunogenic cell death, metastatic tumors also deploy metabolite-centered immune checkpoints, among which tryptophan catabolism and kynurenine signaling represent a prototypical axis of sustained immune suppression.

### 2.4 Tryptophan–IDO1–Kynurenine pathway

The tryptophan catabolism pathway has emerged as a central regulator of immune suppression in the TIME (88–90). Tryptophan, an essential amino acid critical for T-cell proliferation and function, is catabolized primarily through the enzymes indoleamine 2,3-dioxygenase 1 (IDO1) and tryptophan 2,3-dioxygenase (TDO2). In metastatic niches, tumor cells, stromal cells, and tumor-associated immune cells upregulate these enzymes, leading to accelerated degradation of tryptophan into kynurenine (Kyn). The resulting depletion of tryptophan starves effector CD8^+^ T cells, impairing their activation and proliferation, while simultaneously generating immunosuppressive metabolites that reshape the immune landscape. Accumulated kynurenine acts as a potent signaling molecule by activating the aryl hydrocarbon receptor (AhR), a transcription factor that regulates immune tolerance. AhR activation in Tregs enhances their stability and suppressive capacity, while in myeloid-derived suppressor cells (MDSCs) it drives their recruitment and expansion, collectively strengthening the immunosuppressive shield around metastatic tumors. In addition, kynurenine-AhR signaling influences dendritic cells by reducing their antigen-presenting function, further impairing the priming of anti-tumor T-cell responses. Thus, the IDO1/TDO2–kynurenine axis serves as a metabolic immune checkpoint that promotes tumor immune escape (91). Multi-omics studies have provided deep insights into this pathway. Proteomics and metabolomics integration consistently identifies kynurenine as a robust biomarker of immunotherapy resistance. Elevated kynurenine-to-tryptophan ratios (Kyn/Trp) in plasma or tumor tissues correlate with poor prognosis and reduced benefit from PD-1/PD-L1 blockade in melanoma, lung cancer, and hepatocellular carcinoma. Transcriptomic analyses further highlight IDO1/TDO2 expression networks as predictive signatures of immune evasion across multiple metastatic cancer types. Single-cell transcriptomics has revealed heterogeneity in IDO1/TDO2 expression across tumor and stromal compartments, demonstrating that localized tryptophan metabolism can differentially shape immune niches within the same tumor. Clinically, these insights have spurred the development of IDO1 inhibitors such as epacadostat. However, the phase III ECHO-301 trial combining epacadostat with pembrolizumab in melanoma failed to show improved outcomes, raising questions about the complexity of this pathway. Subsequent analyses suggest that the trial design may have overlooked critical factors such as patient stratification, compensatory TDO2 activity, and baseline kynurenine levels (92, 93). Indeed, multi-omics-driven patient stratification appears essential for therapeutic success: patients with high IDO1/TDO2 expression or elevated Kyn/Trp ratios may derive greater benefit from pathway inhibition, while others may require dual blockade strategies (e.g., IDO1 + TDO2 inhibitors) in combination with immune checkpoint inhibitors. Beyond pharmacological inhibition, emerging strategies include targeting downstream AhR signaling to prevent kynurenine-mediated immune suppression, or modulating the gut microbiome to influence systemic tryptophan metabolism and enhance immunotherapy responses (94, 95). The convergence of multi-omics biomarkers, precision trial designs, and combinatorial approaches holds promise for reviving the therapeutic potential of tryptophan pathway modulation in metastatic cancer.

## 3 Multi-omics approaches to decode metabolism–immunity crosstalk

The TIME after metastasis is shaped by intricate metabolic rewiring that directly influences immune dynamics. Traditional approaches, while valuable, often provide only partial insights into these processes. The advent of multi-omics technologies—including metabolomics, proteomics, single-cell sequencing, and spatial omics—has enabled a more comprehensive interrogation of metabolism–immunity crosstalk. By integrating these layers of data, researchers can uncover novel biomarkers, define mechanistic pathways, and generate predictive models that inform therapeutic strategies.

### 3.1 Metabolomics: mapping metabolic shifts

**Figure 3:**
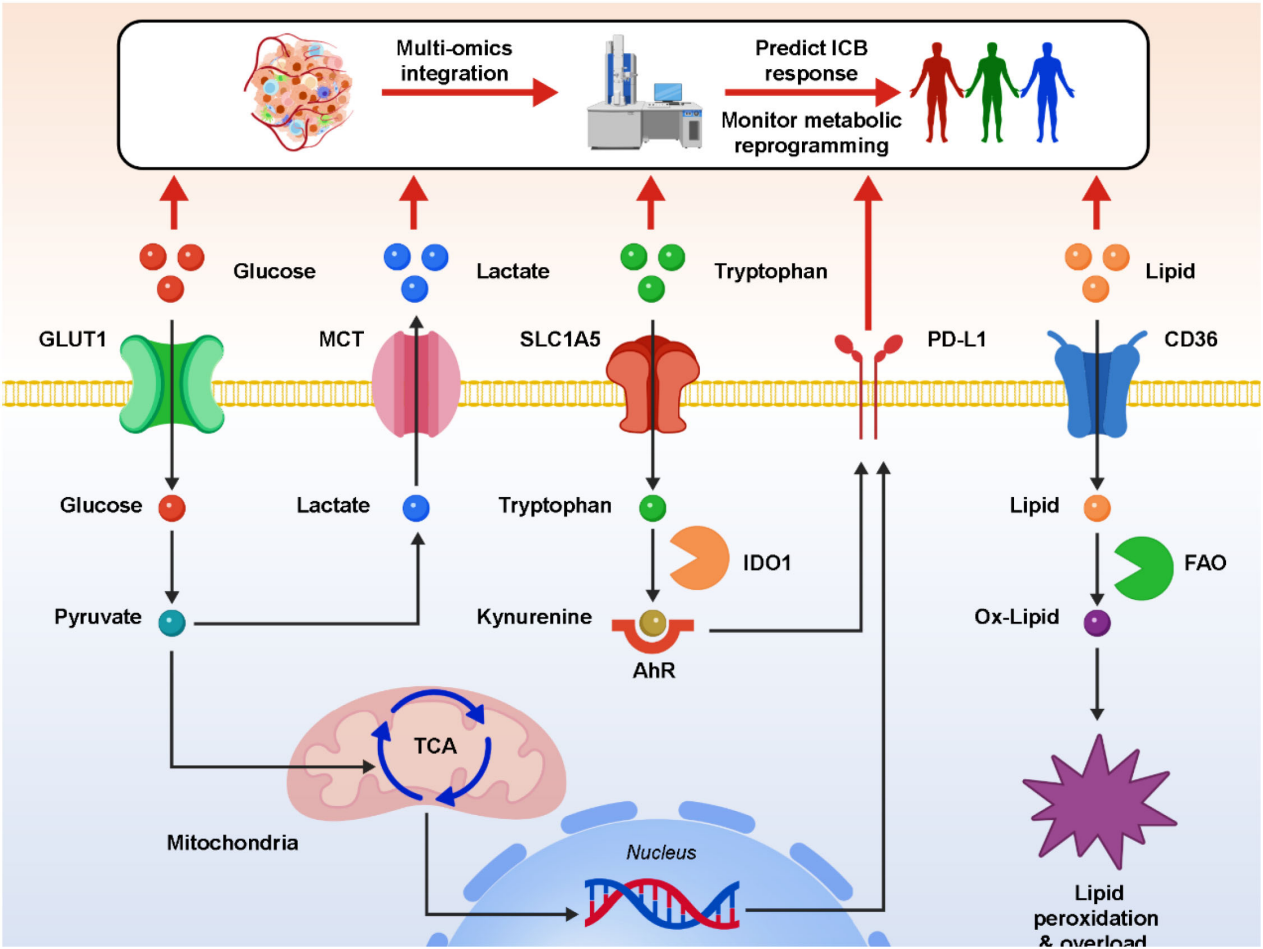
Metabolomics-driven mapping of metabolic reprogramming in the tumor immune microenvironment.

Metabolomics offers a unique window into the functional state of the TIME by directly quantifying small-molecule metabolites that serve as the end products of cellular processes. Unlike genomics or transcriptomics, which capture potential or ongoing molecular programs, metabolomics reflects real-time biochemical activity, making it especially valuable for understanding dynamic changes in metastatic tumors. A variety of analytical platforms are applied to metabolic profiling. Liquid chromatography–mass spectrometry (LC-MS) provides high sensitivity for detecting metabolites across central carbon metabolism, amino acid pathways, and lipid intermediates (96, 97). Gas chromatography–mass spectrometry (GC-MS) is often used for volatile compounds and fatty acid profiling, while nuclear magnetic resonance (NMR) spectroscopy offers reproducibility and quantitative accuracy, albeit with lower sensitivity (98–101). More recently, imaging mass spectrometry (IMS) has enabled the spatial mapping of metabolites within tumor sections, capturing metabolic heterogeneity at near-cellular resolution. In the context of metastatic tumors, metabolomic studies have consistently identified hallmark metabolites associated with immune suppression. These include elevated lactate from aerobic glycolysis, kynurenine from tryptophan catabolism, and lipid intermediates such as long-chain fatty acids and cholesterol derivatives. Elevated kynurenine-to-tryptophan (Kyn/Trp) ratios have been correlated with poor prognosis and reduced benefit from ICB. Similarly, high concentrations of lactate impair CD8^+^ T-cell effector function and are associated with reduced immune infiltration in metastatic niches. Lipid metabolites detected through LC-MS-based lipidomics have also been linked to T-cell exhaustion and dendritic cell dysfunction. Integrative metabolomics with transcriptomic and clinical data has revealed metabolic patterns associated with therapeutic resistance. For example, Enhanced glycolytic flux correlates with PD-L1 upregulation and immune evasion. Increased fatty acid oxidation (FAO) supports tumor survival under stress and drives T-cell dysfunction (102–104). Altered tricarboxylic acid (TCA) cycle activity contributes to immune escape by reshaping cytokine production and stromal signaling. Beyond correlation, metabolomics has provided mechanistic insights into how tumors orchestrate immunosuppression. For instance, metabolite flux analyses show that lactate functions not merely as a by-product but as an immunomodulatory signal, directly driving macrophage polarization. Similarly, kynurenine acts as a ligand for the aryl hydrocarbon receptor (AhR), inducing transcriptional programs that expand regulatory T cells and myeloid-derived suppressor cells. From a translational perspective, circulating metabolites such as lactate, kynurenine, and lipid species represent minimally invasive biomarkers that can be measured in patient plasma or serum. These biomarkers may aid in predicting ICB response, monitoring therapy-induced metabolic rewiring, and stratifying patients for metabolism-targeted interventions. Moreover, longitudinal metabolomics, combined with liquid biopsy approaches, holds promise for real-time therapeutic monitoring in advanced cancer. **Figure 3** illustrates how metabolomics technology identifies tumor-enriched metabolites—including lactate, kynurenine, and lipid derivatives—that drive immune suppression within the TIME. Taken together, metabolomics not only identifies biomarkers of immunotherapy outcomes but also elucidates the mechanistic underpinnings of metabolic–immune crosstalk in the post-metastatic TIME. Its integration with proteomics, transcriptomics, and clinical datasets provides a systems-level framework to discover novel therapeutic targets and to design rational metabolism–immunity combination therapies.

### 3.2 Proteomics and phosphoproteomics

**Figure 4:**
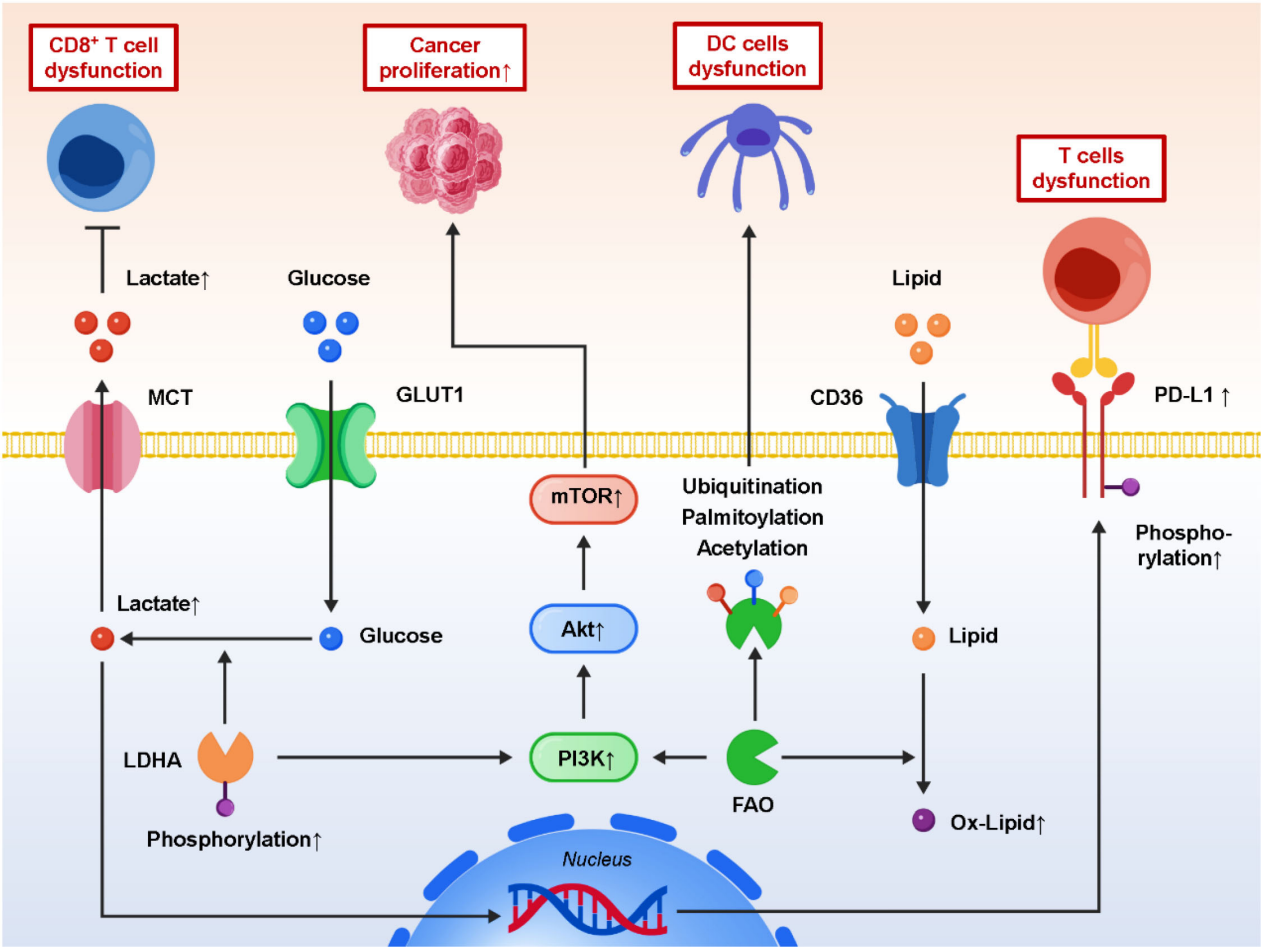
Proteomics and phosphoproteomics reveal how metabolic reprogramming drives immune escape in the tumor immune microenvironment.

Proteomics provides a powerful complement to metabolomics by profiling the abundance and activity states of proteins that govern metabolic and immune pathways (105, 106). Since proteins act as the direct executors of cellular functions, proteomic analyses reveal not only which pathways are upregulated but also how their activity is fine-tuned through post-translational modifications (PTMs). Among these, phosphoproteomics has emerged as a particularly valuable tool, enabling the mapping of phosphorylation events that regulate enzyme activity, metabolic flux, and the stability of immune checkpoint proteins (107–109). For instance, proteomic profiling has demonstrated that phosphorylation of LDHA enhances its enzymatic activity, thereby sustaining high glycolytic flux and lactate accumulation in the TIME. Elevated lactate in turn suppresses CD8^+^ T-cell activity and promotes macrophage polarization toward the M2 phenotype. Likewise, PTMs such as ubiquitination, acetylation, and palmitoylation of enzymes involved in lipid metabolism have been linked to impaired antigen processing and presentation by dendritic cells, weakening the initiation of anti-tumor immunity. Notably, proteogenomic analyses have provided insights into how metabolic enzymes directly regulate immune checkpoint stability. For example, lactate-induced phosphorylation cascades have been shown to enhance PD-L1 stabilization on tumor cells, preventing its proteasomal degradation (110, 111). This finding establishes a direct link between metabolic rewiring and immune evasion, highlighting the potential of targeting metabolic enzyme modifications to sensitize tumors to PD-1/PD-L1 blockade. Beyond single-protein insights, network analyses integrating proteomic and signaling datasets have begun to map the broader connections between metabolic reprogramming and immune pathways. These integrative studies reveal that aberrant metabolic enzyme activity feeds into oncogenic signaling cascades such as PI3K/AKT/mTOR and NF-κB, both of which are key regulators of immune cell survival, differentiation, and cytokine production (112–118). Such analyses underscore the role of proteomics in bridging the gap between metabolism, signaling, and immunity, providing a systems-level understanding of the post-metastatic TIME. From a translational perspective, proteomic and phosphoproteomic datasets can serve as a rich source of biomarkers. Specific PTM patterns on metabolic enzymes or immune regulators may predict responsiveness to immune checkpoint inhibitors (ICIs). For example, phosphorylation signatures associated with LDHA or PD-L1 could identify patients likely to exhibit resistance, guiding the rational design of combination therapies that pair metabolic inhibitors with ICIs (119–121). Furthermore, advances in mass spectrometry sensitivity and clinical proteomics pipelines are paving the way for integrating proteomic biomarkers into routine oncology practice. In summary, proteomics and phosphoproteomics provide critical insights into the regulatory networks that connect metabolic reprogramming with immune modulation in metastatic cancer. By elucidating both protein abundance and modification states, these approaches not only deepen our mechanistic understanding but also open new avenues for biomarker discovery and therapeutic intervention. **Figure 4** demonstrates how proteomic and phosphoproteomic profiling uncovers post-translational modifications of metabolic enzymes (e.g., phosphorylated LDHA) and immune checkpoints (e.g., stabilized PD-L1) that reprogram metabolism and reinforce immune escape.

### 3.3 Single-cell and spatial multi-omics

**Figure 5:**
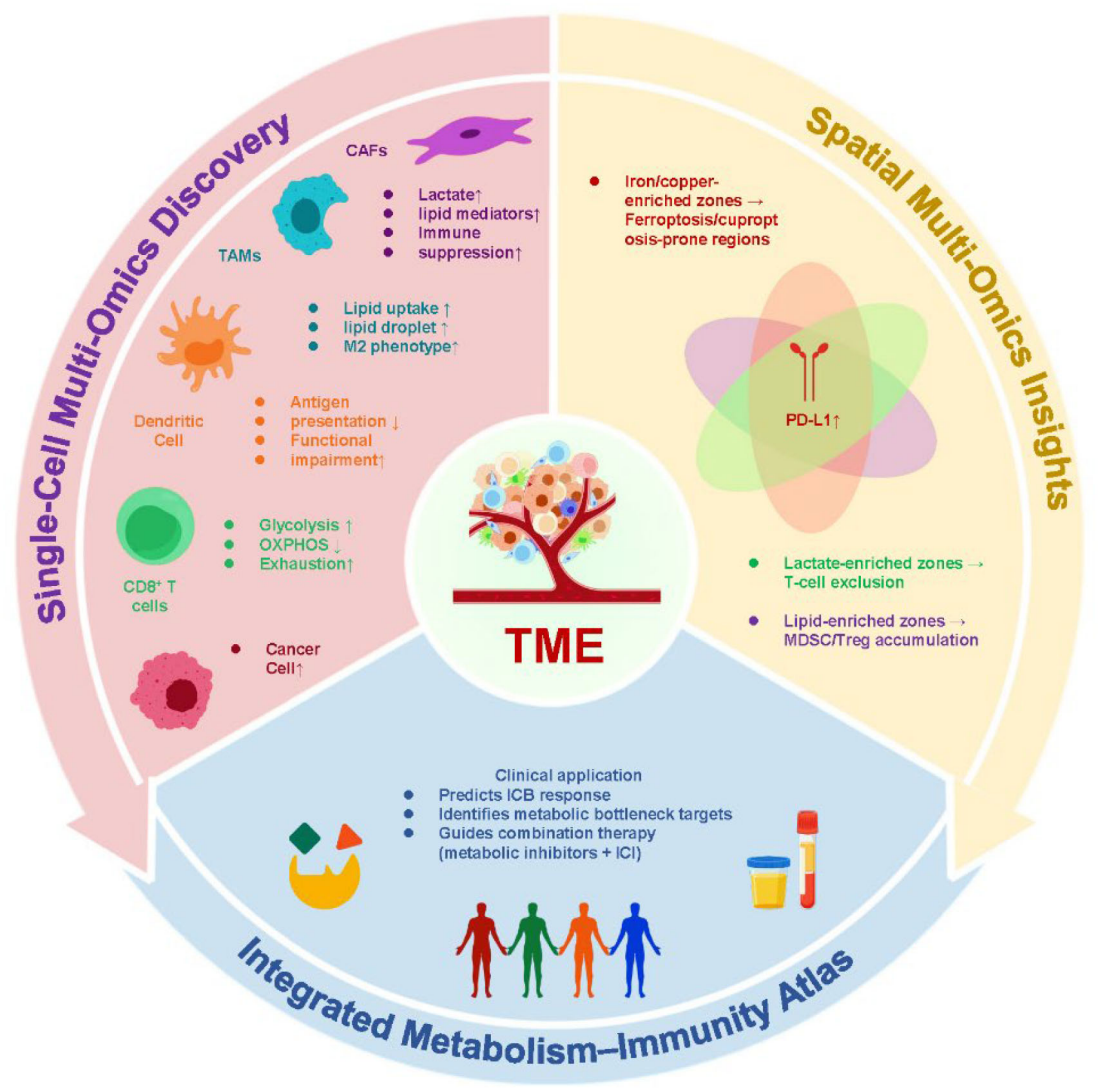
Single-cell and spatial multi-omics reveal the metabolic–immune heterogeneity of the tumor immune microenvironment.

While bulk omics approaches have significantly advanced our understanding of the TIME, they inevitably average signals across mixed cell populations and dissolve spatial structure, thereby masking cell-to-cell heterogeneity—a defining feature of metastatic niches. As a result, bulk assays often cannot distinguish whether a pathway is activated in tumor cells, immune cells, or stromal cells, nor can they resolve where immunosuppressive metabolic “hotspots” are located within the tissue. The advent of single-cell multi-omics technologies has revolutionized this field by enabling the parallel profiling of transcriptomes, epigenomes, and metabolic states at single-cell resolution (122–124). Critically, these approaches add two types of information beyond bulk data: (i) “who” is executing a given metabolic program (cell-type/state specificity) and (ii) how that program co-evolves with immune dysfunction (state coupling). This level of granularity is particularly crucial in metastatic tumors, where metabolic and immune heterogeneity can directly shape immune checkpoint blockade (ICB) sensitivity versus resistance. Single-cell transcriptomics (scRNA-seq) has revealed distinct metabolic programs within specific immune subsets. For example, exhausted CD8^+^ T cells display elevated glycolysis but impaired oxidative phosphorylation, correlating with functional exhaustion and poor responsiveness to ICB (125, 126). In contrast, subsets of macrophages exhibit high lipid uptake and storage, consistent with immunosuppressive polarization. Similarly, cancer-associated fibroblasts (CAFs) have been shown to adopt enhanced glycolytic or lipid metabolic states, fueling immunosuppression by producing lactate and lipid mediators (127–129). Nevertheless, it is important to acknowledge limitations of scRNA-seq–derived computational inferences of metabolism. Transcript abundance does not necessarily reflect enzyme activity, post-translational regulation, substrate availability, or metabolic flux, and pathway-scoring approaches may be biased by dropouts, batch effects, and gene-set selection. Moreover, inferring flux from transcriptional programs typically assumes steady-state behavior and does not capture spatial constraints or cell–cell competition for nutrients. Therefore, scRNA-based metabolic signatures should be interpreted as hypothesis-generating and ideally validated using orthogonal measurements such as single-cell/spatial metabolomics, isotope tracing/fluxomics, and imaging mass spectrometry (IMS). Together, these single-cell profiles move beyond “which pathways are altered” (bulk) to “which cell states drive resistance” (single-cell), enabling the identification of rare but therapy-relevant populations that may dominate clinical outcomes. When combined with single-cell metabolomics, these findings uncover the metabolic fingerprints of individual cell populations, linking biochemical diversity to functional immune phenotypes and providing candidate cell-state biomarkers of immunotherapy failure. Spatial multi-omics approaches provide an additional dimension by preserving the geographic context of metabolic–immune interactions within tumors. Spatial transcriptomics enables the mapping of gene expression patterns across tissue sections, while imaging mass spectrometry (IMS) and spatial metabolomics directly visualize the distribution of metabolites. Importantly, spatial methods add information that single-cell alone cannot fully provide: “where” specific metabolic programs occur and whether they form spatially restricted niches that physically exclude or disable effector immunity. These methods have uncovered lactate-rich and lipid-rich niches that correlate with T-cell exclusion, as well as copper- and iron-enriched microdomains that promote tumor survival and immune evasion. Rather than implying a single universal causal gradient, we emphasize that spatial multi-omics links localized metabolic enrichment with local immune states (e.g., reduced CD8^+^ infiltration, increased suppressive myeloid/Treg programs), thereby pinpointing microregions most likely to drive non-response to ICB. Such spatially resolved insights reveal that metabolic reprogramming is not uniform, but instead generates micro-architectural immune-privileged niches where metastatic tumor cells are protected from immune attack. Building upon these advances, researchers are now developing “metabolism–immune atlases”—integrated frameworks that combine single-cell transcriptomics, metabolomics, proteomics, and spatial mapping. These atlases capture the full complexity of TIME by linking cellular heterogeneity with spatial organization, metabolic flux, and immune states. Such integrated datasets can be leveraged in multiple clinically relevant ways. First, they can guide patient stratification by identifying metabolic–immune signatures predictive of immune checkpoint blockade (ICB) responsiveness. Second, they facilitate the discovery of therapeutic targets, particularly metabolic bottlenecks that are unique to immunosuppressive cell subsets within the metastatic TIME. Third, they can inform the rational design of combination therapies by pinpointing spatially defined microdomains where metabolic inhibition could synergize with immunotherapy. From a translational perspective, single-cell and spatial multi-omics thus hold immense promise for precision oncology. By capturing both cellular heterogeneity and spatial context, these approaches enable more accurate patient stratification and support the development of tailored metabolism–immunity combination strategies. As computational pipelines and clinical-grade platforms continue to evolve, it is anticipated that single-cell and spatially resolved signatures will be incorporated into next-generation diagnostics, empowering clinicians to design dynamic and personalized immunotherapy regimens. Accordingly, **Figure 5** is intended as a conceptual overview illustrating how bulk, single-cell, and spatial approaches differ in what they resolve (“which pathways” vs. “which cells” vs. “where in the tissue”), and how integrating these layers can connect metabolic niches to immune dysfunction and ICB failure.

## 4 Therapeutic implications: linking metabolism and immunotherapy

Understanding the interplay between metabolism and immune regulation in the post-metastatic TIME provides fertile ground for novel therapeutic strategies. Multi-omics studies have not only revealed metabolic vulnerabilities of tumors but also highlighted how these pathways can be exploited in combination with immunotherapy. Here, we discuss four major areas where metabolism-directed interventions intersect with immune checkpoint blockade and precision medicine.

### 4.1 Metabolic targets combined with immune checkpoint inhibitors

One of the most compelling strategies to improve the efficacy of immunotherapy is the targeted inhibition of tumor-driven metabolic pathways that suppress immune responses in the TIME (130–132). From a translational standpoint, multiple metabolism-targeting agents have entered early-phase clinical testing in combination with immune checkpoint inhibitors (ICIs), most prominently those targeting the tryptophan–kynurenine axis (IDO1), arginine metabolism (arginase), and lipid utilization pathways (e.g., FAO/CPT1-related programs) (94, 133). Among these, the glycolytic enzyme lactate dehydrogenase A (LDHA) has been identified as a critical driver of lactate accumulation and microenvironmental acidification. Preclinical models have demonstrated that pharmacological or genetic inhibition of LDHA reduces extracellular lactate levels, restores the cytotoxicity of CD8^+^ T cells, and significantly enhances the antitumor activity of PD-1/PD-L1 blockade (102, 134–136). Similarly, blocking lactate transporters (MCT1/MCT4) prevents lactate export from tumor cells, alleviates immunosuppression, and sensitizes otherwise resistant tumors to immune checkpoint inhibitors (ICIs). Notably, several MCT inhibitors and related strategies are being explored clinically or translationally, reflecting the broad interest in targeting lactate-mediated immune suppression.

Beyond glycolysis, fatty acid oxidation (FAO) represents another key therapeutic axis. In tumor cells, heightened FAO provides metabolic flexibility, sustaining proliferation and survival under stress conditions such as nutrient deprivation and immune attack. However, excessive FAO also enhances immune evasion by increasing the availability of lipid-derived immunosuppressive mediators (137–139). On the immune side, CD8^+^ T cells overloaded with fatty acids become metabolically exhausted, displaying mitochondrial dysfunction and reduced cytokine secretion. Pharmacological inhibition of FAO using etomoxir, a CPT1A inhibitor, has been shown to restore T-cell effector function and synergize with CTLA-4 blockade in preclinical cancer models. However, it is important to note that etomoxir has limitations for clinical translation (e.g., off-target effects and toxicity concerns), highlighting the need for more selective and clinically tractable modulators and for biomarker-guided dosing strategies. Importantly, these findings suggest that selective inhibition of tumor-centric metabolic pathways can improve immunotherapy efficacy without compromising immune cell viability. However, therapeutic windows must be carefully defined, as indiscriminate metabolic inhibition could impair normal immune cell metabolism. In addition to lactate and lipid pathways, arginine metabolism has emerged as a clinically actionable immunometabolic node. Arginase (ARG1/ARG2) activity—often enriched in myeloid suppressor compartments—depletes extracellular arginine, thereby limiting T-cell proliferation and effector programs; accordingly, arginase inhibitors are being evaluated in early clinical studies, frequently in combination with PD-1/PD-L1 blockade (140). Likewise, the tryptophan–IDO1–kynurenine pathway represents one of the most extensively tested metabolic “immune checkpoint” axes (141, 142). Although IDO1 inhibition showed encouraging immunomodulatory signals in early studies, pivotal phase III trials of IDO1 inhibitors combined with anti–PD-1 therapy did not demonstrate added clinical benefit in melanoma, underscoring several key lessons: (i) insufficient biomarker-driven patient selection (e.g., baseline kynurenine/tryptophan ratios or intratumoral IDO1 activity), (ii) incomplete pathway suppression within the tumor microenvironment, (iii) compensatory bypass through related enzymes (e.g., TDO) and downstream AhR signaling, and (iv) context dependence across tumor types and immune ecologies. These experiences argue that future metabolic–immune combinations should incorporate pharmacodynamic confirmation of target engagement, spatial/single-cell readouts of pathway activity, and stratification based on metabolite and immune-state signatures.

To address this challenge, multi-omics studies are increasingly employed to map metabolic dependencies specific to tumor cells versus immune cells, enabling the rational design of interventions that spare beneficial immune populations. Emerging data also support the concept of combination strategies targeting multiple metabolic axes simultaneously. For instance, co-inhibition of LDHA and FAO has been proposed to both reduce lactate-driven immunosuppression and prevent lipid-mediated T-cell exhaustion, thereby enhancing checkpoint blockade efficacy. Furthermore, the development of nanoparticle-based delivery systems may allow the targeted delivery of metabolic inhibitors directly to tumor cells, minimizing systemic toxicity while maximizing therapeutic synergy with ICIs. Importantly, the translational pathway remains non-linear: negative late-stage outcomes (e.g., for IDO1) highlight the need to balance enthusiasm with rigorous trial design, appropriate endpoints, and robust biomarkers.

From a translational perspective, ongoing early-phase clinical trials are evaluating LDHA inhibitors and FAO modulators in combination with PD-1/PD-L1 or CTLA-4 antibodies. More broadly, clinical efforts are also testing arginase inhibitors and other immunometabolic agents alongside ICIs, aiming to relieve nutrient restriction and metabolite-mediated suppression in the TIME. Although most of these trials are still in exploratory stages, preliminary data suggest that metabolic targeting can reprogram the TIME into a more immune-permissive state, expanding the pool of patients who respond to checkpoint immunotherapy. Despite strong preclinical support for targeting tumor metabolism to enhance antitumor immunity, clinical translation has remained challenging. For instance, the phase III ECHO-301 trial of the IDO1 inhibitor epacadostat combined with pembrolizumab failed to show clinical benefit, largely due to the absence of biomarker-driven patient selection, low baseline IDO1/kynurenine activity in many participants, and compensatory TDO2–AhR signaling that sustained immunosuppression despite IDO1 blockade (93). These findings highlight the need for pharmacodynamic confirmation of pathway inhibition and multi-omics–guided stratification of patients with activated tryptophan catabolism. Similarly, early clinical efforts to inhibit lactate metabolism using LDHA inhibitors or MCT1/MCT4 blockers have shown limited progress. Their translation is hampered by systemic toxicity, insufficient tumor-selective delivery, broad expression of MCT transporters in normal tissues, and metabolic plasticity that enables tumors to switch to alternative nutrient pathways (143, 144). These barriers underscore the importance of tumor-targeted delivery systems—such as nanoparticles or engineered exosomes—and combination regimens that co-suppress compensatory metabolic routes. Overall, these clinical experiences indicate that the effectiveness of metabolic–immune interventions depends not only on preclinical mechanistic rationale but also on precise patient selection, validated biomarkers, confirmation of on-target metabolic modulation, and strategies to overcome metabolic redundancy. Integrating multi-omics metabolic signatures and pathway activity scores into clinical trial design will be crucial for improving translational success.

In summary, combining ICIs with metabolic inhibitors such as LDHA and FAO blockers represents a promising therapeutic paradigm. By selectively disrupting tumor-centric metabolic pathways, these strategies aim to overcome immunotherapy resistance and achieve durable responses in metastatic cancer, while acknowledging that clinical success will likely require biomarker-guided patient stratification, confirmation of target inhibition *in situ*, and careful attention to systemic metabolic liabilities.

### 4.2 Exploiting ferroptosis and cuproptosis in immunotherapy

Ferroptosis, an iron-dependent form of regulated necrosis characterized by lipid peroxidation, has emerged as a promising therapeutic mechanism in oncology. Unlike apoptosis, ferroptosis is strongly linked to metabolic reprogramming, as it depends on iron homeostasis, glutathione metabolism, and the activity of antioxidant enzymes such as glutathione peroxidase 4 (GPX4). Inducers of ferroptosis, such as erastin (which inhibits system Xc^-^ transporter) and RSL3 (which directly inhibits GPX4), can trigger tumor cell death in metabolically vulnerable cancers (145–147). Importantly, ferroptotic tumor cells release damage-associated molecular patterns (DAMPs) and lipid-derived mediators, which act as immunogenic signals to activate dendritic cells and promote CD8^+^ T-cell responses. Preclinical models have shown that ferroptosis induction synergizes with PD-1/PD-L1 blockade, overcoming resistance in tumors that are otherwise immune-excluded or immunosuppressed. However, ferroptosis can also be a double-edged sword in the TIME. While inducing ferroptosis in tumor cells is beneficial, excessive lipid peroxidation in immune cells, particularly CD8^+^ T cells, may compromise their persistence and effector capacity. Thus, future therapeutic strategies will need to selectively induce ferroptosis in tumor cells while preserving immune cell viability—a challenge that multi-omics analyses are beginning to address by mapping ferroptosis sensitivity signatures across different cellular compartments. In parallel, cuproptosis—a newly discovered copper-dependent cell death pathway—has attracted increasing attention as a potential immunotherapy adjuvant. Cuproptosis is triggered by intracellular copper accumulation, which drives aggregation of lipoylated proteins in the tricarboxylic acid (TCA) cycle, leading to proteotoxic stress and cell death. The mitochondrial reductase FDX1 has been identified as a key regulator of this process, determining cellular susceptibility to copper-induced toxicity (148–150). Agents such as copper ionophores (e.g., elesclomol) can deliver copper directly into tumor cells, while copper chelators can restore homeostasis in cases of dysregulated copper metabolism. Early evidence suggests that copper-mediated stress not only kills tumor cells but also modulates immune responses by altering cytokine production, antigen processing, and MHC-I expression, thereby reshaping the TIME (151–153). Multi-omics-guided studies are crucial for elucidating these processes. Proteogenomics and transcriptomics have identified ferroptosis-related gene networks (e.g., GPX4, SLC7A11, ACSL4) as predictors of immunotherapy responsiveness, while single-cell RNA sequencing has revealed heterogeneity in ferroptosis sensitivity across tumor and immune subsets. Similarly, integrated metabolomic–transcriptomic analyses have linked cuproptosis-associated genes (FDX1, DLAT, LIAS) with immunotherapy outcomes in cancers such as melanoma, lung cancer, and hepatocellular carcinoma. These insights suggest that ferroptosis and cuproptosis represent not just cell death programs but immune-modulating pathways that can be harnessed for therapeutic benefit. Looking forward, therapeutic strategies may include ferroptosis inducers combined with ICIs, cuproptosis-based agents, or dual targeting of metal-dependent death pathways with immune checkpoint blockade. Precision approaches guided by multi-omics biomarkers will be essential to identify patients most likely to benefit, optimize dosing schedules, and prevent off-target toxicity. The development of nanoparticle-based delivery systems for ferroptosis or cuproptosis inducers may further enhance tumor selectivity and minimize systemic side effects. In summary, ferroptosis and cuproptosis provide novel avenues to potentiate immunotherapy by promoting immunogenic tumor cell death and reshaping the TIME. With the aid of multi-omics analyses and advanced delivery technologies, these strategies may become powerful tools to overcome immune resistance in metastatic cancer.

### 4.3 Targeting IDO1/tryptophan metabolism

The indoleamine 2,3-dioxygenase 1 (IDO1)–kynurenine pathway has long been recognized as a central metabolic immune checkpoint within the TIME. By catalyzing the initial and rate-limiting step of tryptophan degradation, IDO1—and its paralog TDO2 (tryptophan 2,3-dioxygenase)—convert tryptophan into kynurenine (94, 154, 155). This dual effect of tryptophan depletion and kynurenine accumulation drives profound immunosuppressive consequences: effector T cells are deprived of an essential amino acid, impairing their proliferation and cytotoxic function, while kynurenine acts as a potent signaling molecule that activates the aryl hydrocarbon receptor (AhR) in Tregs and MDSCs, enhancing their recruitment, expansion, and suppressive activity. Together, these events foster an immune-tolerant environment that facilitates tumor survival and progression. Because of its central role in immune evasion, the IDO1–kynurenine axis was among the first metabolic pathways targeted clinically in combination with immune checkpoint inhibitors (ICIs). Early-phase trials with epacadostat, a selective IDO1 inhibitor, showed promising results when combined with PD-1 blockade, raising significant enthusiasm (156). However, the phase III ECHO-301 trial (epacadostat + pembrolizumab) in melanoma failed to improve overall survival or progression-free survival, representing a major setback for metabolism-based immunotherapy. Subsequent analyses have provided explanations for this outcome. Tumor cells may compensate for IDO1 inhibition by upregulating TDO2, maintaining kynurenine production. Moreover, not all patients exhibit high baseline kynurenine activity, and the trial lacked robust biomarker-driven patient stratification. This highlights the complexity of tryptophan metabolism in cancer and underscores the importance of integrating multi-omics-derived biomarkers into clinical trial design. For instance, elevated kynurenine-to-tryptophan (Kyn/Trp) ratios, high IDO1/TDO2 expression signatures, and immune infiltration profiles have been identified as predictors of pathway activity and immunotherapy outcomes. Integrating these markers may enable better identification of patient subgroups most likely to benefit from pathway inhibition. Encouragingly, ongoing clinical trials are now exploring next-generation inhibitors that simultaneously target IDO1 and TDO2, or that combine IDO1 inhibition with AhR antagonists, aiming to block downstream signaling. In parallel, dual-targeting strategies that integrate metabolic inhibition with PD-1/PD-L1 or CTLA-4 blockade are under investigation in multiple cancer types, including non-small cell lung cancer, hepatocellular carcinoma, and ovarian cancer. From a translational perspective, multi-omics integration will be critical in guiding these approaches. Metabolomics can quantify circulating kynurenine and tryptophan levels, transcriptomics can profile IDO1/TDO2 expression in tumors and stromal cells, and proteomics can capture regulatory modifications influencing enzyme activity. Combining these layers allows the construction of predictive models that stratify patients according to metabolic–immune signatures. In summary, while the initial clinical trial results were disappointing, the IDO1–kynurenine pathway remains a highly relevant therapeutic target. Future success will depend on leveraging multi-omics-guided biomarker stratification, rational combination strategies, and novel agents that overcome pathway redundancy. Such precision approaches may ultimately unlock the potential of targeting tryptophan metabolism as a means to improve immunotherapy efficacy in metastatic cancer.

### 4.4 Multi-omics guided precision therapy

The profound heterogeneity of the post-metastatic TIME underscores the limitations of uniform therapeutic strategies and highlights the necessity for precision medicine approaches. While traditional clinical biomarkers provide partial information, multi-omics integration offers a holistic framework for stratifying patients based on both metabolic signatures and immune landscapes, thereby enabling tailored therapy design. By combining metabolomics, proteomics, transcriptomics, and single-cell/spatial data, clinicians can identify patient subgroups with distinct metabolic–immune dependencies. For instance, tumors exhibiting high lactate levels and PD-L1 expression may benefit from a regimen combining LDHA inhibitors with PD-1/PD-L1 blockade, whereas patients harboring ferroptosis-sensitive gene signatures (e.g., ACSL4-high, GPX4-low) could respond to ferroptosis inducers in combination with ICIs. Similarly, tumors enriched in FAO-related activity may be susceptible to dual targeting of FAO and immune checkpoints, while those with elevated kynurenine metabolism may require IDO1/TDO2 blockade plus checkpoint inhibition. Recent advances in single-cell and spatial multi-omics have further refined this strategy by allowing the mapping of cell-specific metabolic programs and niche-specific suppressive networks. For example, single-cell RNA-seq integrated with metabolomics can distinguish metabolically exhausted CD8^+^ T cells from active counterparts, while spatial transcriptomics reveals microdomains enriched in lactate or lipids that correlate with immune exclusion (157, 158). These insights enable precision tailoring of therapies—such as deploying copper chelation agents in copper-rich metastatic niches or combining lipid metabolism inhibitors with ICIs in cholesterol-accumulating microenvironments. The translational implications are profound. By constructing multi-omics-driven metabolic–immune atlases, clinicians can design customized therapy combinations for each patient, overcoming resistance mechanisms that are invisible to conventional diagnostics. Integration of machine learning and AI into multi-omics analysis further enhances predictive power, enabling real-time patient stratification and adaptive treatment selection during the course of therapy. Moreover, the incorporation of circulating multi-omics biomarkers (e.g., plasma metabolites, exosomal RNAs, soluble immune checkpoints) holds promise for non-invasive monitoring of therapy response and early detection of resistance. Ultimately, embedding multi-omics insights into clinical decision-making will transform immunotherapy from a “one-size-fits-all” paradigm into a precision-guided strategy, capable of dynamically adapting to the metabolic and immune states of each patient’s tumor. This approach not only promises to increase response rates but also to achieve more durable remissions and improved survival outcomes in patients with advanced and metastatic cancers.

### 4.5 Metabolic biomarkers in clinical trials: current applications and challenges

Metabolic biomarkers are increasingly recognized as essential tools for patient selection, treatment monitoring, and response prediction in immunotherapy-oriented clinical trials (159, 160). Several metabolic signatures have entered early-phase clinical evaluation. For example, the kynurenine-to-tryptophan ratio is widely used as a surrogate marker of IDO1/TDO2 pathway activity, and has been incorporated into clinical studies testing IDO1 inhibitors and AhR antagonists (161, 162). Similarly, lactate-related markers—such as circulating lactate levels, LDH release, and the expression of MCT1/MCT4—have been evaluated to estimate glycolytic burden and immunosuppressive potential within the TIME (163, 164). Tumor tissue–based biomarkers, including FAO-associated gene signatures, mitochondrial activity scores, and glutamine-dependence indices, are also being explored to stratify patients for FAO inhibitors and glutaminase inhibitors (165, 166). Despite these advances, the clinical implementation of metabolic biomarkers faces substantial challenges. First, many metabolic markers exhibit high intra-tumoral and inter-tumoral heterogeneity, making single-point measurements insufficient to capture dynamic metabolic flux. Second, circulating metabolic biomarkers often lack specificity due to contributions from systemic metabolism, inflammation, and non-tumor tissues. Third, metabolic pathways display strong redundancy and compensatory activation, meaning that inhibition of a single metabolic node may not lead to predictable biomarker changes unless accompanied by pharmacodynamic confirmation. Furthermore, the absence of standardized assays, differences in sample handling, and variability in metabolomic platforms complicate cross-trial comparison and broad clinical adoption. To address these limitations, emerging strategies are focusing on multi-omics–based metabolic profiling, spatial metabolomics, and integrated pathway activity scoring systems. These approaches aim to provide more reliable and spatially resolved measurements of metabolic states within the TIME. Additionally, combining metabolic biomarkers with immune profiling—such as PD-L1 expression, T-cell exhaustion markers, or myeloid activation signatures—may improve predictive accuracy for metabolic–immune combination therapies. As clinical trials increasingly incorporate metabolomic endpoints, establishing validated, reproducible, and clinically actionable metabolic biomarkers will be crucial for optimizing patient selection and enhancing the translational success of metabolism-targeted immunotherapies.

## 5 Challenges and future perspectives

Although multi-omics approaches have provided unprecedented insights into the metabolic and immune crosstalk within the post-metastatic TIME, several challenges remain before these findings can be fully translated into clinical practice. To enhance the forward-looking value of this review, we structure the “future perspectives” around three practical questions: (i) how to integrate multi-omics into actionable models, (ii) how to deliver metabolic interventions safely and precisely, and (iii) how to operationalize multi-omics–guided metabolism–immunity combinations in the clinic. Addressing these barriers is critical to harnessing the therapeutic potential of metabolism–immunity interactions.

### 5.1 Technical and computational challenges in multi-omics integration

The integration of metabolomics, proteomics, transcriptomics, and single-cell/spatial omics offers unparalleled opportunities to decode the complex interplay between tumor metabolism and immunity, yet it also introduces formidable technical and computational challenges. From a technical perspective, each omics platform generates data with distinct characteristics. Metabolomics is hampered by metabolite instability, rapid turnover, and variable detection efficiency, often leading to incomplete metabolic coverage (26, 167). Proteomics and phosphoproteomics face difficulties in detecting low-abundance proteins or transient post-translational modifications. Transcriptomics, while high-throughput, does not always reflect actual protein activity, and single-cell/spatial omics approaches add another layer of complexity due to dropout events and technical noise. These discrepancies in sensitivity, dynamic range, and resolution complicate direct integration. Furthermore, sample preparation and batch effects can introduce systematic biases, reducing reproducibility across cohorts and laboratories. On the computational side, multi-omics analysis involves handling high-dimensional, heterogeneous, and noisy datasets. Harmonizing data across omics layers requires advanced pipelines for normalization, feature selection, and dimensionality reduction, while preserving meaningful biological signals. Traditional statistical models are often insufficient to capture nonlinear relationships; instead, network inference, Bayesian modeling, and machine learning approaches are increasingly applied to integrate and interpret data. However, the lack of standardized algorithms and pipelines remains a bottleneck, limiting reproducibility and cross-study comparability. For example, different integration methods (early vs. late integration, matrix factorization, graph-based models) may yield divergent biological conclusions, challenging consensus building. To move beyond general “AI/ML” statements, a key near-term need is to benchmark and standardize representative integration paradigms, including (i) latent factor models (e.g., MOFA/MOFA+), (ii) multi-view clustering and joint feature selection (e.g., iCluster-type frameworks), (iii) supervised multi-omics prediction/feature selection (e.g., DIABLO and other sparse multivariate methods), and (iv) deep generative models (e.g., variational autoencoders) that learn shared embeddings across omics layers. Graph-based learning, including graph neural networks (GNNs), is also increasingly relevant because the TIME is naturally represented as a cell–cell interaction graph with spatial adjacency and ligand–receptor constraints, enabling the integration of spatial omics with signaling and metabolite features. Importantly, the output of integration should be defined clinically (e.g., a parsimonious metabolic–immune signature predicting ICB response, or a ranked list of targetable bottlenecks), rather than only producing descriptive clusters.

Another critical issue lies in cross-cohort integration. Differences in experimental design, sample quality, and data processing pipelines can generate confounding variation that obscures true biological signals. This hampers efforts to validate candidate biomarkers or therapeutic targets across independent datasets. Data harmonization frameworks, federated learning models, and cloud-based repositories are being developed to address these challenges, but widespread adoption remains limited. Finally, there are challenges related to computational infrastructure and scalability. Single-cell and spatial multi-omics alone can generate terabytes of data per study, requiring high-performance computing, optimized storage, and efficient algorithms for real-time analysis. The integration of such datasets with clinical records and imaging data further increases complexity, demanding robust bioinformatics workflows that can bridge the gap between research and clinical application. In summary, although multi-omics integration provides a powerful avenue to map metabolism–immunity crosstalk, it is constrained by technical limitations of detection platforms, computational barriers in data integration, and the absence of standardized, reproducible workflows. Overcoming these challenges will require not only technological innovations but also community-wide efforts to establish shared protocols, open-access datasets, and benchmarking frameworks that ensure robustness and comparability across studies.

### 5.2 Spatiotemporal dynamics of metabolic heterogeneity

Metabolic reprogramming within the TIME is not a static phenomenon but instead evolves dynamically across both spatial and temporal dimensions. These spatiotemporal variations are particularly pronounced in metastatic settings, where tumor cells colonize distinct organ environments and adapt their metabolic strategies to the unique stromal, vascular, and immune landscapes of each niche (168–171). At the spatial level, metastatic lesions exhibit organ-specific metabolic programs. For example, lung metastases often display a glycolytic phenotype with abundant lactate accumulation, shaping an acidified niche that suppresses T-cell infiltration (172). In contrast, liver metastases are enriched in lipid metabolism due to the organ’s intrinsic role in fatty acid and cholesterol handling, fostering an immunosuppressive microenvironment dominated by lipid-accumulating macrophages and dysfunctional T cells (173). Similarly, brain metastases must adapt to a nutrient-restricted and immune-privileged microenvironment, often relying on oxidative phosphorylation and amino acid metabolism for survival. These examples highlight how the metabolic landscape of metastases is organ-specific and tightly coupled with local immune regulation. At the temporal level, tumor metabolism is highly dynamic, influenced by disease progression and therapeutic interventions. As tumors advance, they shift from anabolic programs that support rapid proliferation toward adaptive metabolic states that sustain survival under stress. Therapy-induced remodeling adds an additional layer of complexity: for instance, immune checkpoint blockade may restore T-cell activity but also impose selective pressure that drives tumor cells toward alternative pathways such as fatty acid oxidation or amino acid catabolism. Similarly, targeted therapies may transiently suppress glycolysis but induce compensatory metabolic rewiring that fuels drug resistance. Capturing these spatiotemporal dynamics requires innovative methodologies. Longitudinal sampling of tumors and matched metastatic sites, though clinically challenging, is essential for understanding how metabolic programs evolve under therapy. Advances in spatial multi-omics—including spatial transcriptomics, imaging mass spectrometry, and spatial metabolomics—allow the mapping of metabolites and gene expression *in situ*, revealing microdomains of lactate, lipids, or metal ions that correlate with immune exclusion. In addition, novel imaging-based metabolomic tools, such as hyperpolarized magnetic resonance spectroscopy (MRS) and positron emission tomography (PET) tracers for metabolic flux (e.g., [^18^F]-FDG for glucose, [¹¹C]-acetate for lipids), provide real-time insights into metabolic activity in patients, enabling non-invasive monitoring of metabolic reprogramming. Clinically, acknowledging spatiotemporal metabolic heterogeneity is critical for precision oncology. Biomarkers derived from bulk tumor tissue may not fully reflect metabolic states in metastatic lesions or at later stages of therapy. Thus, incorporating spatially resolved and time-resolved data into clinical decision-making will be vital for guiding patient stratification, predicting therapeutic resistance, and designing dynamic combination strategies. In summary, metabolic heterogeneity in the TIME is both spatially localized and temporally adaptive. Understanding these dynamics through integrated multi-omics and advanced imaging approaches is essential for developing personalized therapeutic strategies that remain effective across the evolving metabolic landscape of metastatic cancer. A key future opportunity is to couple longitudinal liquid biopsy metabolite readouts with periodic spatial profiling or metabolic imaging to capture both systemic and lesion-level dynamics during ICI therapy.

### 5.3 Clinical translation: biomarker validation and patient stratification

Although a growing number of metabolic signatures have been associated with immunotherapy outcomes, their translation into clinically actionable biomarkers faces substantial obstacles. One of the primary challenges lies in the validation gap. Many candidate biomarkers—such as lactate levels, kynurenine-to-tryptophan ratios, ferroptosis-related gene expression, or lipid metabolic profiles—are discovered in preclinical models or small patient cohorts. However, few of these signatures have been rigorously tested and confirmed in large, independent, and multi-center cohorts, which limits their generalizability and clinical utility. Another complicating factor is tumor heterogeneity, both interpatient and intratumoral. A biomarker that is predictive in one cancer type or metastatic site may not apply universally. For instance, kynurenine accumulation may strongly predict resistance in melanoma but show less prognostic power in hepatocellular carcinoma, where lipid metabolism is a more dominant driver of immune escape (174–177). Similarly, intra-tumor spatial heterogeneity means that a biopsy sample may not capture the full metabolic–immune landscape, leading to inconsistent biomarker readouts. In addition, host-related factors such as diet, microbiome composition, comorbidities, and systemic metabolism can influence circulating metabolite levels, further confounding biomarker interpretation. From a practical standpoint, stratifying patients using multi-omics data remains limited by several barriers. Comprehensive profiling involving metabolomics, proteomics, and single-cell/spatial omics is still associated with high costs, specialized equipment, and limited accessibility in routine clinical laboratories. Moreover, the lack of standardized protocols for sample collection, data analysis, and interpretation hinders reproducibility across institutions. Regulatory and ethical challenges—including the approval of multi-omics-based diagnostic assays and integration into electronic health records—further delay translation into practice. Despite these challenges, there are promising avenues to accelerate clinical application. Circulating biomarkers such as plasma lactate, kynurenine/tryptophan ratios, or iron/copper levels can be measured non-invasively and repeatedly, offering potential for real-time monitoring. Exosome-derived noncoding RNAs (ncRNAs), including lncRNAs, circRNAs, and miRNAs, represent another emerging biomarker class, as they capture both tumor and immune cell activity and can be readily detected in liquid biopsies. Furthermore, the development of composite multi-omics signatures—which integrate metabolite ratios, protein modifications, and immune infiltration profiles—may enhance predictive accuracy compared to single-analyte biomarkers. Looking ahead, prospective clinical trials incorporating biomarker-driven patient stratification will be essential. By selecting patients based on validated metabolic–immune signatures, these trials can evaluate metabolism–immunity combination therapies in the most relevant populations, improving the likelihood of clinical benefit. Parallel efforts to standardize assays, develop cost-effective platforms, and incorporate AI-driven predictive models will help overcome current technical and logistical barriers. In summary, while biomarker validation and patient stratification remain key bottlenecks, advances in liquid biopsy technologies, multi-omics integration, and computational modeling hold promise for bridging the gap between discovery and clinical translation. Establishing robust, reproducible biomarkers is crucial to guide personalized metabolism–immunity therapies, ensuring that patients receive the right treatment at the right time. To operationalize this in practice, future trials should predefine biomarker cutoffs, include pharmacodynamic confirmation of target engagement (e.g., metabolite ratios or pathway activity scores), and incorporate on-treatment monitoring to enable adaptive escalation/de-escalation of metabolic co-therapies.

### 5.4 Systemic side effects of metabolic interventions and strategies for their management

While metabolic interventions offer promising avenues for reshaping the TIME, their systemic effects on normal tissues remain a major translational challenge. Many metabolic pathways targeted in tumors—such as glycolysis, fatty-acid oxidation (FAO), glutamine metabolism, and tryptophan–kynurenine catabolism—are also essential for the homeostasis of immune cells, hepatocytes, cardiomyocytes, and skeletal muscle (178, 179). As a result, systemic inhibition of these pathways may lead to unintended toxicities. For example, LDHA inhibitors and MCT1/MCT4 blockers have been associated with skeletal muscle dysfunction, cardiac stress, and red blood cell impairment due to their widespread physiological roles in lactate shuttling (180, 181). Similarly, FAO inhibitors may induce hepatic steatosis or cardiotoxicity, while glutaminase inhibitors can disrupt normal lymphocyte proliferation and gut epithelial repair. Moreover, broad suppression of IDO1/TDO2 signaling can alter microbial metabolism and systemic immune tolerance, raising additional safety considerations. Several strategies have been proposed to mitigate these systemic toxicities and enhance the therapeutic window of metabolic interventions. First, tumor-targeted delivery platforms—including nanoparticles, liposomes, polymeric micelles, and engineered exosomes—can enhance intratumoral drug accumulation while sparing normal tissues. Second, intermittent or adaptive dosing schedules may reduce metabolic pressure on healthy organs while maintaining antitumor efficacy. Third, combination strategies that use lower doses of multiple metabolic inhibitors, rather than high-intensity blockade of a single pathway, may minimize toxicity by avoiding excessive metabolic bottlenecks in normal tissues. Fourth, biomarker-guided patient selection—based on metabolic dependency signatures, enzyme expression levels, or real-time metabolic flux measurements—can ensure that only patients with high metabolic pathway activation receive the corresponding inhibitors. Lastly, emerging approaches such as tissue-specific pro-drugs and allosteric modulators hold potential for achieving more selective metabolic modulation with fewer systemic effects. As the field advances, integrating toxicity monitoring, pharmacodynamic assessment, and metabolic imaging into clinical trial design will be essential to ensure safe and effective deployment of metabolic-immune therapies. Addressing these systemic challenges will be critical for translating metabolic interventions from preclinical promise to durable clinical benefit.

### 5.5 Future directions: toward precision metabolism–immunity therapies

The convergence of emerging technologies and interdisciplinary approaches is paving the way for a new era of precision therapies that bridge metabolism and immunity. While current challenges in biomarker validation, data integration, and therapeutic translation remain formidable, several promising avenues are rapidly evolving to overcome these barriers and transform multi-omics insights into clinically actionable strategies. To strengthen the strategic depth of this section, we outline three forward-looking pillars: AI-enabled multi-omics integration, precision delivery systems (nanoparticles/exosomes), and a practical clinical translation roadmap for multi-omics–guided combination therapy.

AI-driven multi-omics integration represents a cornerstone of this transformation. By harnessing artificial intelligence and machine learning, researchers can integrate high-dimensional datasets from metabolomics, proteomics, transcriptomics, and spatial/single-cell omics into unified predictive models. These approaches not only improve the identification of hidden metabolic–immune interactions but also enable the development of real-time patient stratification tools. For example, deep learning models trained on multi-omics and clinical data can predict patient response to immune checkpoint blockade, highlight metabolic vulnerabilities unique to each tumor, and inform dynamic treatment adjustments during therapy (182–186). In practice, current AI toolkits for integration span (i) representation learning/generative models (e.g., autoencoders and variational autoencoders) that learn shared latent embeddings from multi-omics, (ii) multimodal attention models that weight omics layers according to clinical relevance, and (iii) graph learning (GNNs) that integrate spatial neighborhoods, ligand–receptor links, and cell–cell communication with metabolic features. A key translational requirement is interpretability: models should not only classify responders vs. non-responders but also output human-readable drivers (e.g., ranked pathways, targetable enzymes/transporters, or spatially localized risk niches) and quantify uncertainty to support clinical decision-making. Federated learning offers another future avenue by enabling model training across institutions without moving patient-level data, which may accelerate multi-center validation while preserving privacy.

Advanced delivery platforms, such as nanoparticle- and exosome-based systems, provide innovative methods for targeted modulation of the TIME. Nanoparticles can encapsulate small-molecule inhibitors, siRNAs, or CRISPR-based gene-editing tools directed against metabolic enzymes, delivering them selectively to tumor or stromal cells while sparing immune effectors. Similarly, engineered exosomes can serve as natural carriers for immunomodulatory RNAs or metabolic inhibitors, exploiting their intrinsic tropism for specific cell types (187–189). These delivery technologies promise to minimize systemic toxicity and maximize therapeutic efficacy, thereby expanding the feasibility of metabolism–immunity combination therapies in clinical settings. Specifically, nanoparticles offer high payload capacity, controlled release, and tunable surface ligands for cell-type targeting (e.g., tumor cells vs. myeloid suppressor cells), whereas exosomes offer favorable biocompatibility and endogenous trafficking properties. However, key challenges remain, including off-target biodistribution (especially liver/spleen sequestration), potential immunogenicity, heterogeneity of particle/exosome preparations, limited cargo-loading efficiency for some payloads, and scale-up/quality-control constraints under GMP manufacturing. For metabolic interventions, these platforms are particularly attractive for delivering (i) siRNA/ASOs against enzymes/transporters (e.g., LDHA, MCTs, IDO1, ARG1), (ii) inhibitors with narrow systemic therapeutic windows, and (iii) combinatorial payloads that co-target metabolic suppression and immune activation.

The concept of theranostics is another emerging frontier. By integrating diagnostics and therapy within a single platform, theranostic agents can both monitor metabolic activity in real time and simultaneously modulate immune responses. For instance, radiolabeled probes can visualize lactate flux, amino acid metabolism, or copper accumulation via PET/MRI imaging, while coupled therapeutic agents act to normalize these pathways or enhance immunotherapy responsiveness. This dual functionality enables dynamic and personalized cancer management, where treatment decisions are guided by continuous metabolic monitoring. Next-generation clinical trial designs will be essential to bring these innovations into practice. Adaptive trial frameworks that incorporate multi-omics biomarkers, liquid biopsy monitoring, and real-time imaging can flexibly stratify patients, evaluate novel drug combinations, and rapidly refine therapeutic regimens (190–192). Basket trials that group patients by shared metabolic–immune signatures—rather than cancer type—may accelerate the validation of targeted interventions. In parallel, international collaborations and consortia focused on multi-omics standardization will help establish reproducible biomarkers and regulatory guidelines for precision metabolism–immunity therapies. Looking forward, the integration of AI-driven analytics, advanced delivery platforms, theranostic tools, and adaptive clinical trials holds the potential to transform immunotherapy from a partially effective approach into a highly personalized, dynamic treatment paradigm. By tailoring therapies to the evolving metabolic and immune landscapes of metastatic tumors, these strategies may ultimately achieve the long-sought goal of durable responses and improved survival for patients with advanced cancers.

In summary, while significant obstacles remain in data integration, heterogeneity mapping, and clinical application, the convergence of multi-omics technologies, AI analytics, and novel therapeutic platforms holds great promise. By bridging metabolism and immunity in the post-metastatic TIME, future strategies can move beyond single-pathway targeting to develop combinatorial, personalized, and dynamically adaptable therapies, ultimately improving outcomes for patients with advanced cancer.

## 6 Conclusion

Metabolic reprogramming has emerged as a fundamental driver of immune modulation within the post-metastatic TIME. Key alterations—including enhanced glycolysis and lactate accumulation, dysregulated lipid metabolism, metal-dependent cell death programs such as ferroptosis and cuproptosis, and the tryptophan–IDO1–kynurenine pathway—synergistically contribute to the establishment of an immunosuppressive niche. These changes not only sustain tumor proliferation and metastasis but also underlie therapeutic resistance. Importantly, such metabolic shifts are not isolated events; rather, they are deeply interwoven with immune-regulatory networks, collectively dictating the outcomes of immunotherapy and shaping patient prognosis. The advent of multi-omics technologies—including metabolomics, proteomics, single-cell sequencing, and spatial multi-omics—has created unprecedented opportunities to decode metabolism–immunity crosstalk with unparalleled resolution. By integrating multi-layered datasets, researchers can delineate tumor- and immune-specific metabolic signatures, identify predictive biomarkers of therapeutic response, and uncover novel metabolic vulnerabilities that can be therapeutically exploited. This integrative approach positions multi-omics as a critical translational bridge that connects mechanistic insights from basic science with precision applications in clinical oncology. Looking forward, the convergence of metabolism-targeted therapies with immunotherapy represents a transformative frontier. Combining metabolic inhibitors with immune checkpoint blockade, leveraging ferroptosis or cuproptosis inducers to enhance tumor immunogenicity, and modulating amino acid metabolism to reverse immune tolerance all represent promising avenues to overcome resistance and broaden the spectrum of patients who benefit from immunotherapy. Moreover, the incorporation of multi-omics-guided biomarkers, AI-powered analytics, and advanced delivery systems (such as nanoparticles and engineered exosomes) will further enable the design of personalized, dynamic, and adaptive treatment paradigms. In conclusion, decoding the metabolism–immunity interplay through multi-omics not only deepens our mechanistic understanding of metastatic cancer biology but also lays the foundation for next-generation combination therapies. Such integrative strategies hold the promise of enhancing the efficacy of immunotherapy, extending patient survival, and ultimately improving the quality of life for individuals facing advanced malignancies. Importantly, multi-omics–guided personalized metabolism–immunotherapy is poised to become a central direction of future oncology, offering a framework in which metabolic–immune signatures inform customized therapeutic decisions. Achieving this vision will require closer interdisciplinary collaboration among oncologists, immunologists, computational biologists, and bioengineers to accelerate biomarker validation, optimize therapeutic design, and translate multi-omics discoveries into clinically actionable practice. Together, these collaborative and technology-driven efforts will usher in a new era of precision, patient-centered metabolism–immunity therapeutics.
